# Maximizing the extraction yield of plant gum exudate using response surface methodology and artificial neural networking and pharmacological characterization

**DOI:** 10.1038/s41598-023-37847-x

**Published:** 2023-07-06

**Authors:** Shazia Noureen, Sobia Noreen, Shazia Akram Ghumman, Sami A. Al-Hussain, Huma Hameed, Muhammad Anwar-Ul-Haq, Ali Irfan, Fozia Batool, Muhammad Umair Hassan, Samina Aslam, Magdi E. A. Zaki

**Affiliations:** 1 Institute of Chemistry, University of Sargodha, Sargodha, 40100 Pakistan; 2 College of Pharmacy, University of Sargodha, Sargodha, 40100 Pakistan; 3 Department of Chemistry, College of Science, Imam Mohammad Ibn Saud Islamic University (IMSIU), Riyadh, 11623 Saudi Arabia; 4 Faculty of Pharmaceutical Sciences, University of Central Punjab, Lahore, 54000 Pakistan; 5 Department of Physics, University of Sargodha, Sargodha, 40100 Pakistan; 6 Department of Chemistry, Government College University Faisalabad, Faisalabad, 38000 Pakistan; 7 Department of Chemistry, The Women University Multan, Multan, Pakistan

**Keywords:** Chemical biology, Computational biology and bioinformatics, Drug discovery, Plant sciences, Diseases, Oncology, Chemistry

## Abstract

*Prunus armeniaca* gum is used as food additive and ethno medicinal purpose. Two empirical models response surface methodology and artificial neural network were used to search for optimized extraction parameters for gum extraction. A four-factor design was implemented for optimization of extraction process for maximum yield which was obtained under the optimized extraction parameter (temperature, pH, extraction time, and gum/water ratio). Micro and macro-elemental composition of gum was determined by using laser induced breakdown spectroscopy. Gum was evaluated for toxicological effect and pharmacological properties. The maximum predicted yield obtained by response surface methodology and artificial neural network was 30.44 and 30.70% which was very close to maximum experimental yield 30.23%. Laser induced breakdown spectroscopic spectra confirmed the presence Calcium, Potassium, Magnesium, Sodium, Lithium, Carbon, Hydrogen, Nitrogen and Oxygen. Acute oral toxicity study showed that gum is non-toxic up to 2000 mg/Kg body weight in rabbits, accompanied by high cytotoxic effects of gum against HepG2 and MCF-7cells by MTT assay. Overall, Aqueous solution of gum showed various pharmacological activities with significant value of antioxidant, antibacterial, anti-nociceptive, anti-cancer, anti-inflammatory and thrombolytic activities. Thus, optimization of parameters using mathematical models cans offer better prediction and estimations with enhanced pharmacological properties of extracted components.

## Introduction

Natural gums are magnetizing more scientific attention due to their structural and compositional diversity and functional properties, as well as their ease of alteration. There is a growing market for plant gums in pharmaceutics, cosmetics, and textile sector. So search for novel plant-based gums with desired functional properties is subject of increasing interest^1^ because choosing a new biopolymer source for application needs comprehensive understanding of physicochemical and functional properties. Characterization of these polymers give a chance to industries for selection of an appropriate option according to their particular requirement without avoiding extensive trial and error^2,3^. These characteristics are sensitive to separation techniques and can be appreciably modified by adopted drying method^4^. Plant gums are well-known natural biopolymers possessing variety of pharmacological activities like antioxidant, antimicrobial, anti-nociceptive, and anti-inflammatory^5^. These gums can undergo easy chemical modifications to overcome some shortcomings like microbial contaminations, shelf life, thickening, uncontrolled hydration and viscosity changes and meet commercial realities^6^.

*Prunus armeniaca* L. (Apricot) from Rosaceae, is extensively distributed in Asia. The *Prunus armeniaca* gum (PAG), acquired from its branches is commonly used as a food additive^7^. In Iran it is considered a whitening agent, coughs reliever and improves eye sight^8^. It is an anthelmintic, expectorant and antidote substance. It is also known as an antioxidant, stabilizer, and emulsifier. Various modalities have been opted to formulate therapeutic formulation of PAG^9^. Composition of a gum changes with species and geographical conditions. Hence, investigating indigenous specie for gums has a key role to search for better candidates with variety of applications^10^.

Recently developed extraction strategies have opened a new window of opportunities for extraction and isolation of bioactive constituents from nature embedded sources with enhanced efficacy and efficiency. Additionally, these approaches have an advantage of minimizing the required time, solvent quantity, and temperature over conventional technique^11^. Extraction efficiency is significantly controlled by selected method, solvent quantity, temperature, and solid to liquid ratio individually or in combination. Obtaining optimal conditions for extraction using conventional one-factor methods is a tough and tiresome task. Limitations of a one-factor conventional method can be prevailed over by employing empirical approaches^12^. These mathematical models offer a technical source and theoretical support to get a combination of optimized conditions saving resources, time, and energy improving quality and quantity of extraction^13^. Response surface methodology (RSM) is a combination of statistical methods for planning experiments, creating models, analyzing the effects of variables, and looking for the ideal circumstances. It is used to evaluate the effect of several variables simultaneously and analyze the relationship between independent variables and response variables^14^. Artificial neural network (ANN) is a mathematical tool that simulates the human brain. It is considered a superior choice than RSM because of its self-training capability to perform a multi-factor response optimization which enhances the reliability of prediction. This model mimics the human nervous system working with a plenty of neurons. Neurons are arranged in layers forming input layers, many hidden layers, and an output layer. Input layers transfer signals to output layers via hidden layers^15^. During the recent past, application of ANN in extraction have proved it a suitable and advanced tool for effective optimization and accurate prediction for non-linear relation of an extraction method^16^. The ANN model has a wide range of applications in the domains of chemical engineering, hydrology, and food technology, due to its extraordinary flexibility and competence in data fitting, optimization, and prediction^17^. In the present work RSM and ANN both empirical models were used to get the optimal conditions for extraction yield of PAG.

## Materials and methods

### Materials

The *Prunus armeniaca* gum (PAG) was collected from tree trunk from Malakwal, Punjab, Pakistan in August 2021. The collected gum along with plant parts was taxonomically identified at Department of Botany University of Sargodha by taxonomist Dr. Amin Ullah Shah (Associate Professor). A voucher specimen (No.UOS-PA-21-18) was deposited in the herbarium, University of Sargodha, Sargodha for further reference. All the procedures of collection and identification of plant material were in accordance with the guidelines of National Herbarium of Pakistan, Flora of Pakistan, and International Plant Name Index. All other reagents and chemicals used were commercially available and of analytical grade. Chemical used in experimental work include ethyl alcohol, sodium alginate: Mw 216, 2, 2- diphenyl-1-picrylhydrazyl (DPPH), Muller Hinton agar were obtained from Sigma-Aldrich and potassium dihydrogen phosphate was procured from Merck. All other chemicals and reagents used are commercially available and analytical grade.

### Extraction of PAG

**Figure 1:**
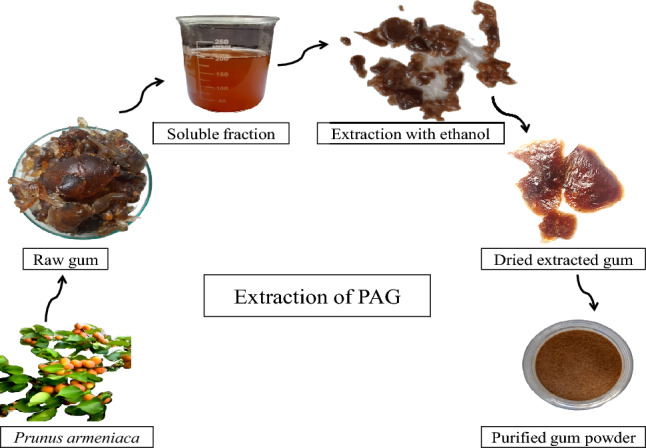
Schematic diagram for extraction of purified PAG from raw gum obtained from trunk.

Extraction and purification method of PAG was used as reported earlier with minor modifications^18^. Raw gum was cleaned, ground and dissolved in distilled water on a magnetic stirrer for a particular time at 160 rpm speed. Gum suspension was kept all night at 4 ˚C to hydrate completely. Insoluble portion was separated by passing through a muslin cloth. Separated solution was centrifuged at 3000×*g* for 30 min and suspended particles were removed. Soluble fraction was precipitated with ethanol. Precipitated gum was dried at room temperature, ground, sieved through 80-mesh sieve and kept in airtight viols for further analysis. Brief description of the extraction method is given in Fig. 1.

1 $$Yield\,\, \left(\% \right)= \frac{Mass \,\,of\,\, purified\,\, PAG}{Mass \,\,of\,\,crude\,\, PAG}\times 100,$$

### Statistical modeling and data calculation

#### Response surface methodology

The RSM was applied to obtain most appropriate combination of various parameters to get maximum output from applied extraction method. A three-level four-factor central composite design (CCD) was applied for statistical analysis^19^. A total of 29 runs were executed to appraise the effect of independent parameters. Previously reported literature and preliminary trials were used as source for selection of independent variables. Effect of various conditions on extraction yield was evaluated by varying extraction temperature (X_1_ = 25–35 °C), extraction pH (X_2_ = 6.1–6.8), extraction time (X_3_ = 4–6 h), and gum/solvent ratio (X_4_ = 1:10–1:30 w/v). The percent yield was calculated in triplicate and mean values were used for regression analysis. Experimental values obtained were evaluated with software package Design-Expert version (13, Minneapolis, MN, USA) to predict a set of optimized conditions for best results of extraction. The 3D response surface and 2D contour plots were obtained from RSM to find the conditions for optimized yield. Additionally, these plots were used to analyze the combined effect of applied parameters and correlation between parameters. Extraction yield values were put into a second-order polynomial equation for development of an empirical model correlating yield response and selected parameters.

2 $$ Y = b_{0} + b_{1} X_{1} + b_{2} X_{2} + b_{3} X_{3} + b_{4} X_{4} + b_{11} X_{1}^{2} + b_{22} X_{2}^{2} + b_{33} X_{3}^{2} + b_{44} X_{4}^{2} + b_{1} b_{2} X_{1} X_{2} + b_{1} b_{3} X_{1} X_{3} + b_{1} b_{4} X_{1} X_{4} + b_{2} b_{3} X_{2} X_{3} + b_{2} b_{4} X_{2} X_{4} + b_{3} b_{4} X_{3} X_{4} + E, $$

where Y is the output response (yield %), X_1_ is extraction temperature, X_2_ is extraction pH, X_3_ is extraction time, and X_4_ is gum/solvent ratio. The b_o_ is the intercept, b_1_-b_4_ are the coefficient of linearity. B_11_, b_22_, b_33_, and b_44_ are the quadratic coefficients. B_1_b_2_, b_1_b_3_, b_1_b_4_, b_2_b_3_, b_2_b_4_, and b_3_b_4_ are the coefficient of interactions. E is the error function of the empirical model^20^.

#### Artificial neural networking

ANN is a statistical technique to predict a nonlinear relationship between output response and input parameters^21^. The same parameters used in RSM were analyzed by ANN software (STATISTICA 10). Constructed ANN model was trained, applied, and authenticated using experimental extraction yield acquired by the 29 experimental runs. The first input layer consist of four independent parameters; extraction temperature (X_1_ = 25–35 °C), extraction pH (X_2_ = 6.1–6.8), extraction time (X_3_ = 4–6 h), and gum/solvent ratio (X_4_ = 1:10–1:30 w/v). The middle hidden layer comprises by six neurons to optimize the input parameters for maximum yield percent. The third output layer is constituted by one dependent response (yield percent)^15^.

### Elemental analysis of PAG by laser induced breakdown spectroscopy (LIBS)

Micro and macro-elemental composition of PAG was determined by using laser induced breakdown spectroscopy (LIBS). For this purpose, the finely dried PAG powder was pressed into pellets sizes of 5 mm and 15 mm by hydraulic press. Briefly, the PAG pellet was ablated by focusing the laser beam obtained from a Q-switched Nd: YAG laser (Energy = 230 mJ; λ = 355 nm and repetition rate = 10 Hz using a convex lens (focal length = 25 cm) on the PAG pellet (target). The sample was mounted on the holder; multiple laser pulses were shot on it with continuous rotation of target to prevent the crater formation on pellet surface. The spectrum was obtained by multi-channel spectrometer, LIBS2500 + (Ocean Optics, USA) and data was transferred to computer by using OOILIBS-plus software for detailed elemental analysis^22^.

### Ex vivo mucoadhesive ability

Mucoadhesive ability of PAG was examined by preparing microbeads of PAG with alginate using ex vivo wash off method. Blank microbeads of sole alginate (F1 = standard) and PAG-alginate (F2 = 1:2, F3 = 1:1) were prepared using our previously described method^9^. Fresh goat intestinal mucosa was purchased from slaughter house and a piece of 2 cm × 2 cm area was cut for further use. Intestinal mucosa piece was tied on a glass slide of area 7.5 cm × 2.5 cm using thread. About 50 microbeads were separated and positioned onto the wet goat intestinal mucosa. Prepared slides were hanged into USP disintegration test apparatus having 900 mL of pH 1.2 and 7.4 separately, operated with regular up and down movements. After a particular time, interval movement was stopped and attached microbeads were counted^23^.

### Toxicological analysis of PAG

Acute oral toxicity of PAG was determined according to Animal Research: Reporting of In Vivo Experiments (ARRIVE) guidelines. Whole experimental protocols were approved by Biosafety and Ethical Review Committee of University of Sargodha via reference no (Ref: SU/ORIC/394/22/09/2022). Briefly, healthy albino rabbits (1–1.5 kg) were kept for a week in animal house in clean cages under 12 h light/dark cycle for acclimatization with a standard diet and water supply. Animals were randomly divided into control and treated groups (n = 3). Treated group was orally administered 2000 mg/kg body weight of PAG mixed in water while control group received only food and water. Animals were observed once daily for signs of illness and mortality for 14 days. Body weights, food and water intake was checked on 1st, 7th and 14th day. At the end of trial period, blood samples were collected for hematological and biochemical studies. Lastly, rabbits were sacrificed via anesthetic intravenous overdose of sodium pentobarbital and vital organs were removed for histological observations. Collected organs were weighted and preserved in 10% v/v solution. Tissues were cut to prepare slides which were observed under microscope^24,25^.

### Pharmacological properties of PAG

#### Antioxidant activity

Antioxidant activity of PAG was evaluated by 2, 2- diphenyl-1-picrylhydrazyl (DPPH) free radical assay and Ferric reducing antioxidant power (FRAP) assay. For DPPH assay Butylated hydroxyl anisole was used as standard. PAG powder was dissolved in distilled water to prepare a series of pre decided concentrations. About 3.00 mL of gum solutions were mixed with 0.5 mL of DPPH solution in methanol (0.1 mM) and kept for 30 min at 37 °C. Absorbance of solution was measured at 517 nm using UV–VIS spectrophotometer (Shimadzu, Japan). Absorbance of standard solutions was measured in the same way. The inhibition percent was calculated using formula mentioned in below equation^26^:

3 $$Inhibition\,\, \left(\%\right)= \frac{{Abs}_{1}-{Abs}_{2}}{{Abs}_{1}}\times 100,$$

For FRAP assay 1.00 mL of PAG solution was mixed with 3.5 mL of phosphate buffer (0.2 M and 6.6 pH). Then 2 mL of 1% potassium ferricyanide solution was added. Mixtures were incubated at 37 ± 1 °C for 20 min. Then 2.5 mL of 10% trichloroacetic acid was added to stop the reaction. All the sample mixtures were centrifuged at 3000 rpm for 10 min and supernatant was separated. About 2.5 mL of supernatant was mixed with 2.5 mL of distilled water and finally 0.5 mL of ferric chloride (0.1%) was added. Absorbance of samples and ascorbic acid used as standard was measured at 700 nm^27^.

#### Antibacterial activity

The antimicrobial activity of PAG was determined using disk diffusion method against 4 different but most important bacterial strains *Staphylococcus aureus* (ATCC 25923), *Escherichia coli* (ATCC 25922)*, Pseudomonas aeruginosa* (ATCC 2853) *and Haemophilus influenza* (ATCC 49247)^28^. The chosen bacterial strains were cultured in Muller Hinton agar medium. Solution of agar was prepared dissolving 34 g in one liter distilled water. Whole assembly including agar solution, petri plates, and filter paper discs were sterilized at 121 °C in an autoclave for 30 min. Agar solution was spread uniformly in petri plates and allowed to cool. After solidification, petri plates were inverted to stop water droplets to avoid hindrance in microorganism growth. 10 µL of selected bacterial strain suspensions were spread on prepared medium using micropipette. Petri plates were gently rotated to spread evenly. Discs were loaded with PAG aqueous solution (1%) and put on agar surface. Plates were kept for 24 h at 37 ± 0.5 °C. Results were calculated in mm of inhibition zones.

#### Anti-nociceptive activity

##### Hot plate test analysis

Animals were divided in 4 groups (n = 5). Group 1 was considered as negative control (Saline), Group 2 was considered as positive control (Diclofenac sodium, 50 mg/kg), Group 3 was treated with PAG (200 mg/kg) and Group 4 was treated with PAG (400 mg/kg). All animals were placed one by one into a glass beaker on the heated plate (50 ± 0.5 °C) for 40 s (maximum time). During this analysis, the reaction time was noted during 0, 30, 60, 90, and 120 min post-treatment of saline, diclofenac sodium and PAG, when the mice licked their paws^29^. The inhibitory activity was calculated according to below mentioned equation:

4 $$Inhibition\,\,\left(\mathrm{\%}\right)= \frac{{T}_{n}-{T}_{o}}{{T}_{o}}\times 100,$$

where Tn is the reaction time after the administration Diclofenac sodium (50 mg/kg) (positive control) or PAG (200 mg/kg) or PAG (400 mg/kg) and T_0_ was the initial reaction time.

##### Writhing test analysis

Second method for detecting the anti-nociceptive (analgesic) activity of PAG was carried out by writhing count analysis induced by acetic acid. Overnight fasted mice with free access to water were used and divided into 4 groups (n = 5). 1.00% acetic acid (10 mL/kg) was given to all 4 groups (1 control and 3 treated groups) via intra-peritoneal route to induce writhes (contraction of abdominal muscles). 2.5 h’ pre-administration of saline was done to group 1 (considered as negative control) and 2.5 h pre-administration of Diclofenac sodium (50 mg/kg) was done to group 2 (considered as treated with positive control). In case of treated groups, PAG (200 mg/kg) to group 3 and (400 mg/kg) to group 4 was given out 2.5 h before the administration of acetic acid to access the analgesic activity of aqueous solution of PAG, by counting the numbers of writhes after 5 min of I.P injection of 1% acetic acid solution and continued for up to 1 h^30^. A reduction in number of writhes was linked to the analgesic activity of PAG vs. the control group. That was counted by below mentioned equation.

5 $$Inhibition \,\,of\,\, writhes\,\, \left(\%\right)= \frac{{W}_{c}-{W}_{t}}{{W}_{c}}\times 100,$$

where W_t_ is the average number of writhes in treated groups and Wc is the average number of writhes in the control group.

#### Anti-inflammatory activity

Anti-inflammatory effect of PAG was evaluated by the carrageenan induced paw edema assay. Each group containing n = 5 (Swiss Albino mice) and total groups were five (1 negative control with normal saline, 1 positive control with Diclofenac sodium, 1 with phlogistic agent that induced inflammation and 2 with PAG (200 mg/kg and 400 mg/kg). PAG groups were administered by PAG through oral route via syringe. Diclofenac sodium (50 mg/kg) was injected I.P. to positive control group. Further, 50 ul of carrageenan solution (1%) was injected to the plantar surface of the left hind paw to all animals of the five groups after 30 min of treatment of diclofenac sodium to group 2 and PAG to group 4 and 5. The potential anti-inflammatory effect of PAG was evaluated with a digital plethysmometer by measuring the paw volume of each animal after each hour of total 4 h of experimental study^31^.

#### Thrombolytic activity

A series of solution (0.2, 0.4, 0.6, 0.8 and 1%) was prepared by dissolving PAG powder in distilled water by stirring on magnetic stirrer for 15 min. All solutions were filtered to get clear solution by 0.22-micron syringe filter paper. Commercially available Streptokinase was used as standard. A vial of 15,00,000 I.U. was purchased and mixed with 5 mL distilled water. Whole blood (5 mL) was taken from albino rabbits of weight 1–1.5 kg and transferred in sterilized, labeled, pre weighted vials (n = 3) and incubated at 37 °C for 45 min for clot formation. Weight of clot was determined after removing serum carefully. About 1 mL of PAG solutions and streptokinase standard was added to clot containing vials and kept aside. After 90 min fluid was decanted and vials were reweighted. Percentage thrombolysis was calculated using formula^32,33^

6 $$Thromobolysis\,\, \left(\%\right)= \frac{Final \,\,weight\,\, of\,\,clot}{Initial\,\, weight\,\, of\,\,clot}\times 100,$$

#### Cell viability analysis

A new drug candidate require assessment linked to cell viability analysis in normal and cancer cell environment to comprehend their biomedical applications for future purposes. The in vitro cytotoxicity of PAG was evaluated by MTT assay at different concentration on the HepG2 and MCF-7 cell lines for cancerous environment and on the Vero, cell lines for the normal cell environment. DMEM (Dulbecco’s Modi-field Eagle medium) along with 10% FBS (Fetal Bovine serum) was used for culturing the all-cell lines. The cells were seeded in 96 well plates and incubated for 48 h at 37 °C in a 5% CO_2_ atmosphere. The test samples were solubilized in 500 µg/ ml of DMSO (dimethyl sulfoxide) with further dilution by water up to 500 µg/ml. At the end remaining test samples were frozen for later use. Ultimate frozen concentration of 500 µg/ml was thawed and further diluted in sequential concentrations of PAG gels (50, 100, 150, 200, 300, and 400 µg/ml). Incubation of all sequential concentration was done up to 48 h with culture plates. After above procedure, addition of 100 µl of MTT (5 mg/ml) was carried out to each well with additional 4 h incubation. Subsequent removal of the culture media with PBS and 0.2 ml of DMSO was also done in each well. The absorbance was checked via a micro plate reader at 550 nm (Thermo Fisher Scientific, Rockfold, IL, USA)^7,34,35^.

### Statistical analysis

All the experimental studies were carried out in triplicates and analysis was performed using STATISTICA 10 and Design-Expert 13 software.

### Declaration of ethical statement for animal study

Animals selected for study were handled in strict accordance with the recommendations of “Guide for the Care and Use of Laboratory Animals”. The whole experimental procedure was further approved by Biosafety and Ethical Review Committee of University of Sargodha via reference no (Ref: SU/ORIC/394/22/09/2022). The animal study is reported in accordance with ARRIVE guidelines (https://arriveguidelines.org).

## Results and discussion

### Extraction of PAG

The quality of an extracted material is affected by a number of factors for instance, selected plant part, solvent, extraction method, extraction temperature and pH and, solid to liquid ratio etc. From laboratory to industrial scale all the influencing factors are controlled and optimized during extraction^11^. Soluble fraction of PAG was separated from crude gum by distilled water which was precipitated using ethanol, dried, and powdered. Extraction conditions were optimized by RSM and ANN molding. Optimization of extraction parameters facilitated the achievement of maximum yield. Purified PAG was lighter in colour probably due to leaching of the polyphenols during precipitation^36^. The obtained gum was light brown, odorless, rough, and irregular in shape. It formed light golden sticky solution in water.

### Optimization of extraction process

In the present study optimization of extraction yield was performed using RSM and ANN model. Structural design of ANN to get predicted values is given in Table 1a. The independent variables temperature, pH, extraction time, and PAG/water ratio were quantitatively evaluated to appraise their effect on dependent response (yield). The Table1b depicts a comparative view of dependent variable extraction yield and predicted values of RSM-CCD and ANN for all 29 runs. Extraction yield of PAG was shown in the range from 23.4 to 30.23%. Results reveal that predictive results of RSM and ANN are in good agreement with experimental results but predictive ability of ANN was found superior than RSM. ANN is considered a versatile predictive technique which uses different number of hidden neurons to obtained best suited predictive ability.

**Table 1:** (a) Structural design of ANN applied to construct model (b) Central composite design of four independent parameters with experimental and predicted values of percentage yield.

(a) ANN specifications							
No of neurons in input layer	4						
No of neurons in hidden layer	6						
No of neurons in output layers	1						
Training error	0.020893						
Test error	0.001040						
Hidden activation	Exponential						
Output activation	Tanh						
No of neurons in input layer	4						

(b) Run no	Parameters*				Yield (%)		
	X_1_ (°C)	X_2_	X_3_ (h)	X_4_ (w/v)	Experimental	RSM pred.	ANN pred.
1	35	6.4	6	10	27.30	25.92	26.99
2	45	6.4	4	20	26.60	27.24	27.24
3	45	6.4	5	20	29.20	28.06	28.73
4	45	6.4	6	10	25.90	26.47	25.10
5	35	6.1	6	20	23.40	24.03	23.52
6	35	6.8	6	20	27.30	26.10	27.01
7	25	6.4	4	10	27.20	27.12	27.21
8	25	6.4	5	10	27.60	28.18	27.19
9	25	6.8	5	20	27.70	28.23	27.78
10	45	6.4	6	30	30.00	29.01	28.35
11	25	6.8	4	20	27.70	27.41	27.15
12	35	6.4	6	30	25.30	27.05	26.15
13	25	6.4	6	20	30.10	30.44	30.27
14	25	6.4	6	20	30.23	30.44	30.15
15	25	6.1	4	20	24.54	24.51	24.37
16	25	6.4	5	20	30.10	29.45	29.95
17	25	6.1	6	20	27.30	26.51	26.94
18	25	6.1	6	10	24.80	24.97	24.73
19	25	6.4	6	20	30.20	30.44	30.15
20	45	6.8	6	20	26.20	27.03	26.32
21	35	6.4	4	20	26.40	25.91	26.95
22	25	6.4	6	20	30.10	30.44	30.70
23	25	6.8	6	10	28.50	28.62	28.63
24	25	6.4	6	30	29.70	29.06	29.74
25	35	6.4	5	20	26.10	26.77	26.99
26	25	6.8	6	30	27.20	27.18	27.24
27	25	6.4	6	30	29.40	29.06	29.73
28	25	6.4	4	30	27.30	27.51	27.10
29	45	6.4	6	20	28.90	28.98	28.45

*(X_1_ = 25–35 °C), extraction pH (X_2_ = 6.1–6.8), extraction time (X_3_ = 4–6 h), and gum/solvent ratio (X_4_ = 1:10–1:30 w/v).

The ANOVA results of RSM are described in Table 2. The R^2^ value of model for yield is high (0.86) indicating the well fitting of model for second-order polynomial equation. The value of p (0.007), CV % (3.60), adjusted R^2^ (0.73), mean (27.66), and adequate precision (8.94) point to the suitability of model. Correlation between independent parameters and yield (%) was evaluated. The maximum yield (30.23%) was calculated in run 14 at temperature = 25 °C, pH = 6.4, extraction time = 6 h, and PAG/water ratio = 1:20 w/v. The fitted second-order polynomial Eq. (7) for extraction yield of PAG is shown below.

7 $$ {\text{Actual Yield }}\left( \% \right) \, = {29}.{54} + 0.{\text{93X}}_{{1}} + {2}.{\text{88X}}_{{2}} - 0.{\text{82X}}_{{3}} + 0.{\text{24X}}_{{4}} - 0.{\text{16X}}_{{1}} {\text{X}}_{{2}} - 0.{\text{28X}}_{{1}} {\text{X}}_{{3}} - 0.{5}0{\text{5X}}_{{1}} {\text{X}}_{{4}} - 0.0{\text{9X}}_{{2}} {\text{X}}_{{3}} - 0.{\text{41X}}_{{2}} {\text{X}}_{{4}} + 0.{7}0{\text{2X}}_{{3}} {\text{X}}_{{4}} - {2}.{\text{86X}}_{{1}}^{2} + 0.{\text{32X}}_{{2}}^{2} + {1}.{\text{98X}}_{{3}}^{2} - {1}.{\text{24X}}_{{4}}^{2} . $$

**Table 2:** ANOVA analysis of RSM.

Source	Sum of squares	df*	Mean square
Model	87.24	14	6.23
X_1_-temperature	1.49	1	1.49
X_2_-pH	1.10	1	1.10
X_3_-extraction time	0.1766	1	0.1766
X_4_-PAG/water ratio	0.0852	1	0.0852
X_1_ X_2_	0.2589	1	0.2589
X_1_ X_3_	0.0128	1	0.0128
X_1_ X_4_	2.59	1	2.59
X_2_X_3_	0.0225	1	0.0225
X_2_X_4_	0.6058	1	0.6058
X_3_ X_4_	0.1479	1	0.1479
X_1_^2^	17.14	1	17.14
X_2_^2^	32.10	1	32.10
X_3_^2^	0.0088	1	0.0088
X_4_^2^	7.44	1	7.44
Residual	13.91	14	0.9935

*Degree of freedom.

p-value = 0.0007, Mean = 27.66, R^2^ = 0.86, C.V% = 3.60

Adeq precision = 8.94.

**Figure 2:**
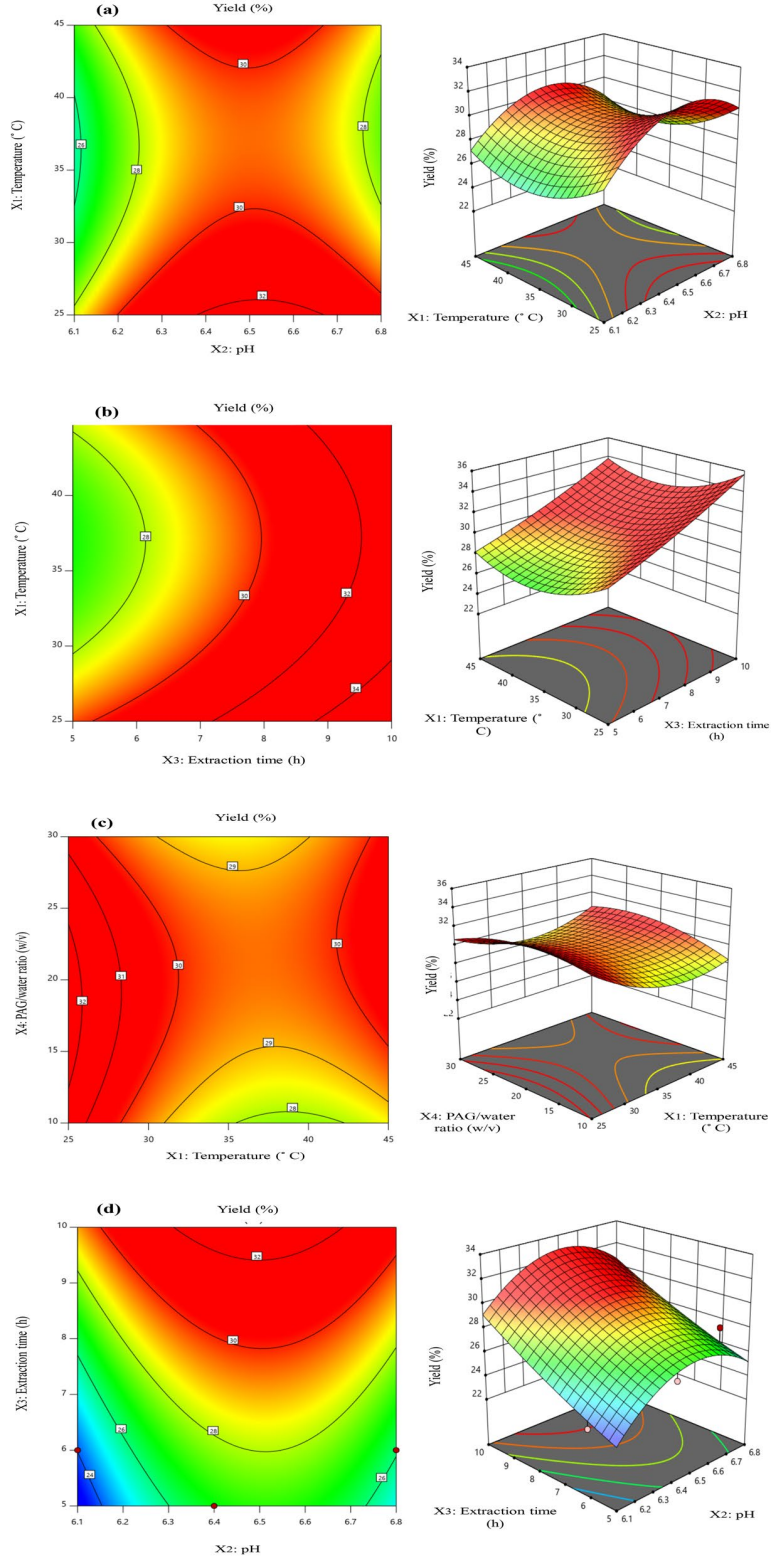

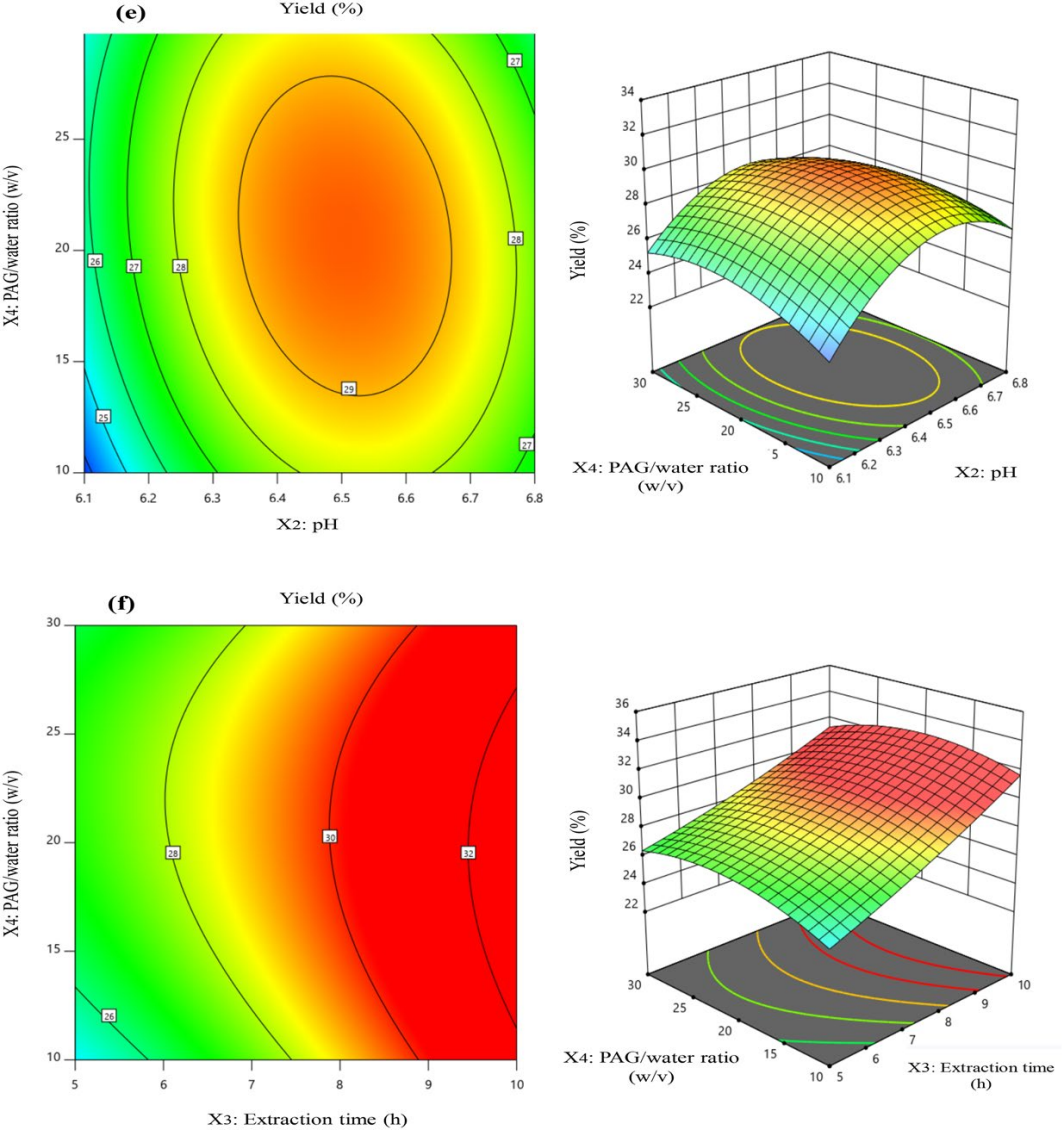
2D contour graphs and 3D response surface plots for: (**a**) X_1_ = Temperature and X_2_ = pH, (**b**) X_1_ = Temperature and X_3_ = Extraction time, (**c**) X_1_ = Temperature and X_4_ = PAG/water ratio, (**d**) X_2_ = pH and X_3_ = Extraction time, (**e**) X_2_ = pH and PAG/water ratio, (**f**) X_3_ = Extraction time and PAG/water ratio.

The 3D response surface and 2D contour plots reveal the correlation between output response (% yield) and selected independent parameters. Figure 2a explains the effect of temperature and pH on the extraction yield kept extraction time and PAG/water ratio constant. Figure 2b represents the effect of temperature and extraction time on the yield while pH and PAG/water ratio is constant. Figure 2c depicts the influence of temperature and PAG/water ratio. Figure 2d demonstrates the effect of pH and extraction time. Figure 2e shows effect of pH and PAG/water ratio. Figure 2f explains the effect extraction time and PAG/water ratio.

### Elemental analysis of PAG by LIBS

**Figure 3:**
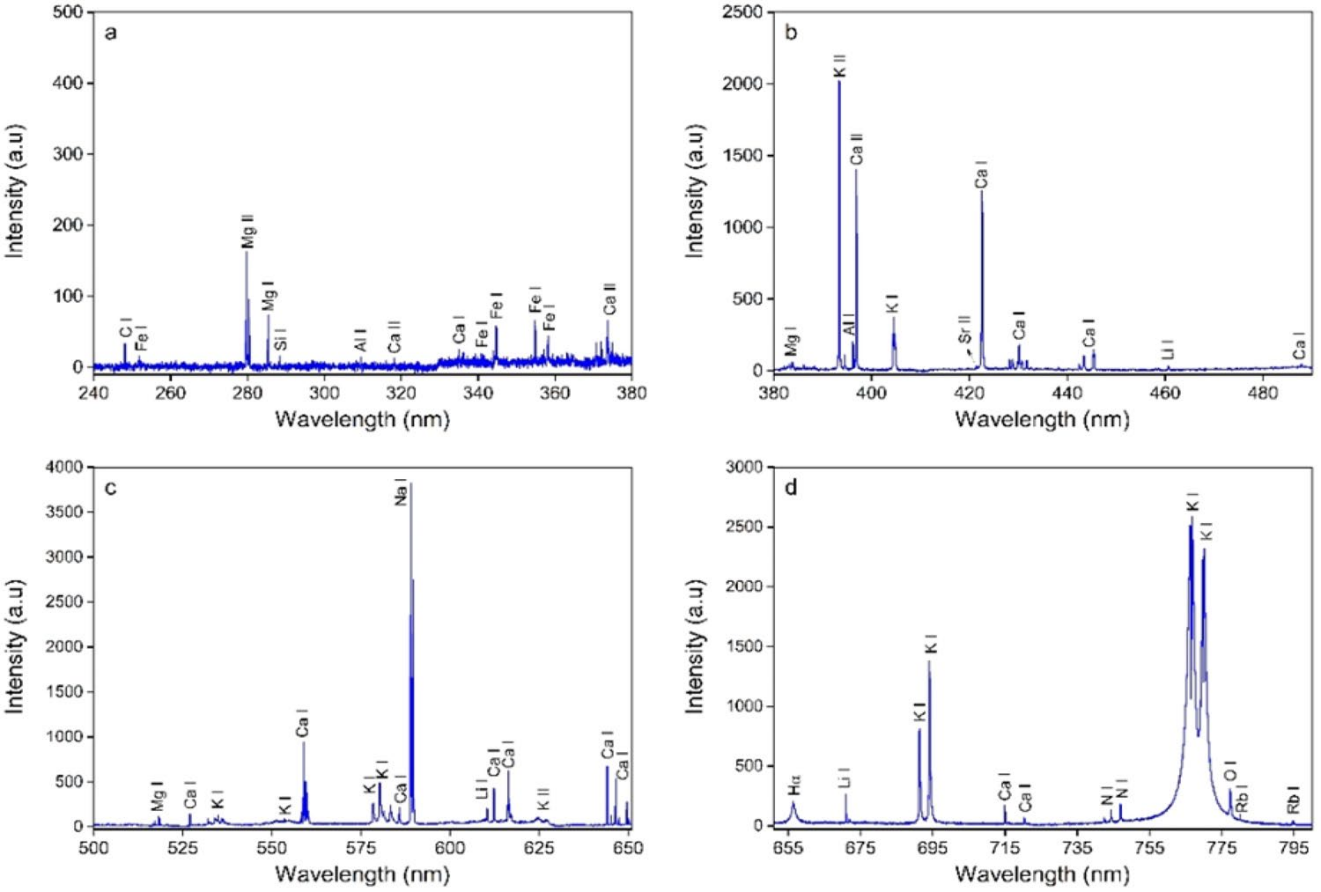
LIBS spectra of *Prunus armeniaca* gum (PAG) in the wavelength range of 240–800 nm. Elements detected in PAG are also mentioned against the corresponding transitions.

The LIBS spectra of PAG pellets were recorded in the range of 230–805 nm as shown in Table 3. The spectra were recorded at various points of the target to detect the major elements with laser energy of 25 mJ. The spectra obtained have a varying degree of spectral atomic and ionic lines which were correlated to the element abundance in PAG sample. For the identification of the spectral lines obtained and characteristic elements presence, NIST data base was used^37^. The spectra shown in Fig. 3 clearly confirms the presence of Calcium (Ca), Potassium (K), Magnesium (Mg), Sodium (Na), Lithium (Li), Carbon (C), Hydrogen (H), Nitrogen (N) and Oxygen (O) in PAG sample, similar to already reported literature^18^.

**Table 3:** The wavelengths corresponding to elements identified in PAG by LIBS analysis.

Species	Wavelength (nm)
Ca I	422.67, 428.30, 428.93, 429.90, 430.25, 430.77, 431.86, 442.54, 443.50, 445.48, 527.03, 534.95, 558.20, 558.88, 559.45, 559.85, 585.75, 612.22, 616.21, 616.95, 643.91, 644.98, 646.26, 647.16, 649.38, 649.96
Ca II	370.60, 373.69, 396.85
Mg I	285.21, 383.23, 383.83, 516.73, 517.27, 518.36
Mg II	279.55, 280.43
K I	404.41, 404.72, 578.23, 580.17, 581.21, 583.18, 691.11, 693.88, 766.49, 769.90
Al I	394.40, 396.15
Na I	588.99, 589.59
Li I	460.28, 610.35, 670.79
H	656.28
N I	742.36, 744.22, 746.83
O I	777.41, 777.53
C I	247.86

### Ex vivo mucoadhesive ability

**Figure 4:**
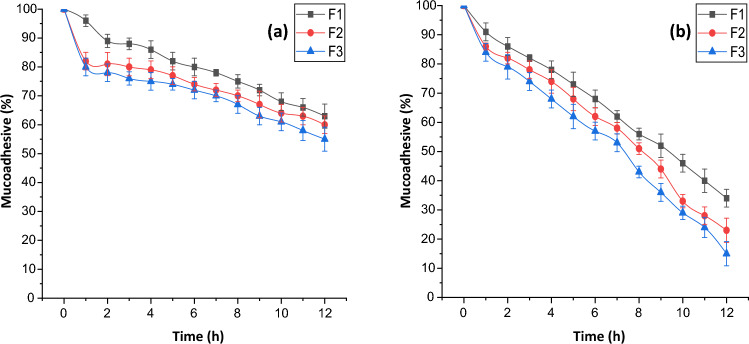
Mucoadhesive properties of microbeads of PAG, (**a**) pH 1.2 buffer (**b**) pH 7.4 buffer.

Natural gums are chemically polysaccharides equipped with enormous hydroxyl and carboxyl groups in their polymeric structure. These hydrogen bond forming groups makes them mucoadhesive in nature^38^. Mucoadhesive ability of PAG by formulating its microbeads with alginate was studied on to a goat intestinal mucosa in pH 1.2 and 7.4 using wash off method. It is evident from results shown in Fig. 4 that washing off speed of beads was considerably high in pH 7.4 than that of pH 1.2. Lower mucoadhesive ability in pH 7.4 buffer is attributed towards the degradation of calcium ions^39^.

### Toxicological analysis of PAG

#### General observations

After the end of trial period of 14 days, there were no sign of toxicity and illness observed during physical examination. All the animals were healthy and active. Body weight, food and water intake showed in Table 4 have no significant difference in control and treated group. These observations indicate that PAG is non-toxic up to 2000 mg/kg body weight in rabbits.

**Table 4:** General observation for acute toxicity and vital organs weight in control and PAG treated groups of rabbits.

General observations	Control group	Treated group
Mortality	Nil	Nil
Sign of illness	Nil	Nil
Body weight (g)		
Pretreatment	1285 ± 54	1148 ± 89
Day 1	1289 ± 55	1144 ± 88
Day 7	1300 ± 53	1151 ± 92
Day 14	1315 ± 49	1157 ± 86
Water intake (mL)		
Pretreatment	127 ± 7.50	124 ± 7.57
Day 1	132 ± 9.29	122 ± 7.93
Day 7	136 ± 10.81	128 ± 4.16
Day 14	142 ± 11.71	133 ± 6.55
Food intake (g)		
Pretreatment	147 ± 9.64	127 ± 3.6
Day 1	150.34 ± 6.8	122.67 ± 6.8
Day 7	155 ± 6.42	126 ± 4.72
Day 14	164.67 ± 7.23	134 ± 4.04
Vital organs weight (g)		
Liver	36 ± 1.5	35 ± 0.5
Stomach	17.8 ± 1.1	16.7 ± 1.2
Heart	2.8 ± 0.2	3.6 ± 0.3
Kidney	4.1 ± 0.2	3.9 ± 0.46
Spleen	0.41 ± 0.08	0.34 ± 0.04

#### Histological analysis

**Figure 5:**
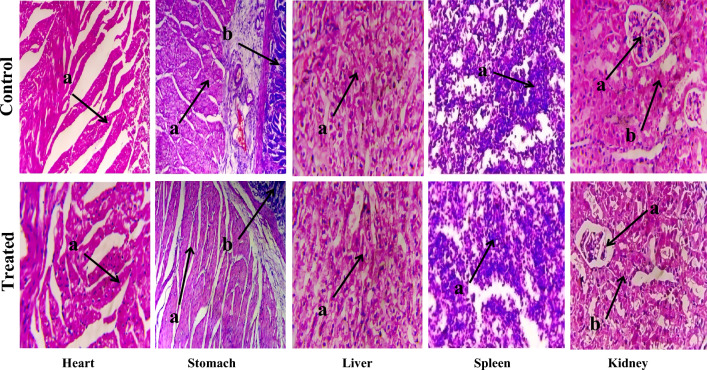
Histological evaluation of control and PAG treated group. Heart: (**a**) cardiac muscle fibers; Stomach: (**a**) muscularis (**b**) mucosa; Liver: (**a**) hepatocytes; Spleen: (**a**) red pulp; Kidney: (**a**) glomerulus (**b**) renal tubules.

Absolute weight of various organs obtained from control and treated group was determined and shown in Table 4. Vitol body organ weight of both control and treated group was very close to each other. No histological changes were seen in liver, heart, stomach, spleen, and kidney as shown in Fig. 5.

#### Hematological and biochemical analysis

Hematological and biochemical analysis was performed to check any difference in both groups and results are listed in Table 5. All parameters of blood chemistry, lipid profile, liver profile and renal profile were comparable in both groups and within reference range^24^.

**Table 5:** Hematological and biochemical analysis of rabbits.

Parameters	Control group	Treated group
Hematology		
Hb (g/dl)	13.5 ± 0.3	13.8 ± 0.85
Total WBCs (103/µl)	10.5 ± 0.15	10.1 ± 0.72
RBC (106/µl)	5.73 ± 0.87	5.1 ± 0.34
Platelets (103/µl)	150.7 ± 6.02	147.3 ± 4.04
Liver profile		
AST (U/L)	20.7 ± 3.51	22.3 ± 5.5
ALT (U/L)	79 ± 9.84	64 ± 5.56
ALP (U/L)	33.7 ± 8.32	38.4 ± 7.09
Renal profile		
Creatinine (mg/dl)	0.67 ± 0.25	0.8 ± 0.26
Urea (mg/dl)	22 ± 3.46	19.6 ± 1.52
Uric acid (mg/dl)	3.7 ± 0.56	3.9 ± 0.72
Lipid profile		
Cholesterol (mg/dl)	103 ± 6.55	94 ± 7.93
Triglyceride (mg/dl)	118 ± 4.35	115.3 ± 4.16

### Pharmacological properties of PAG

#### Antioxidant

**Figure 6:**
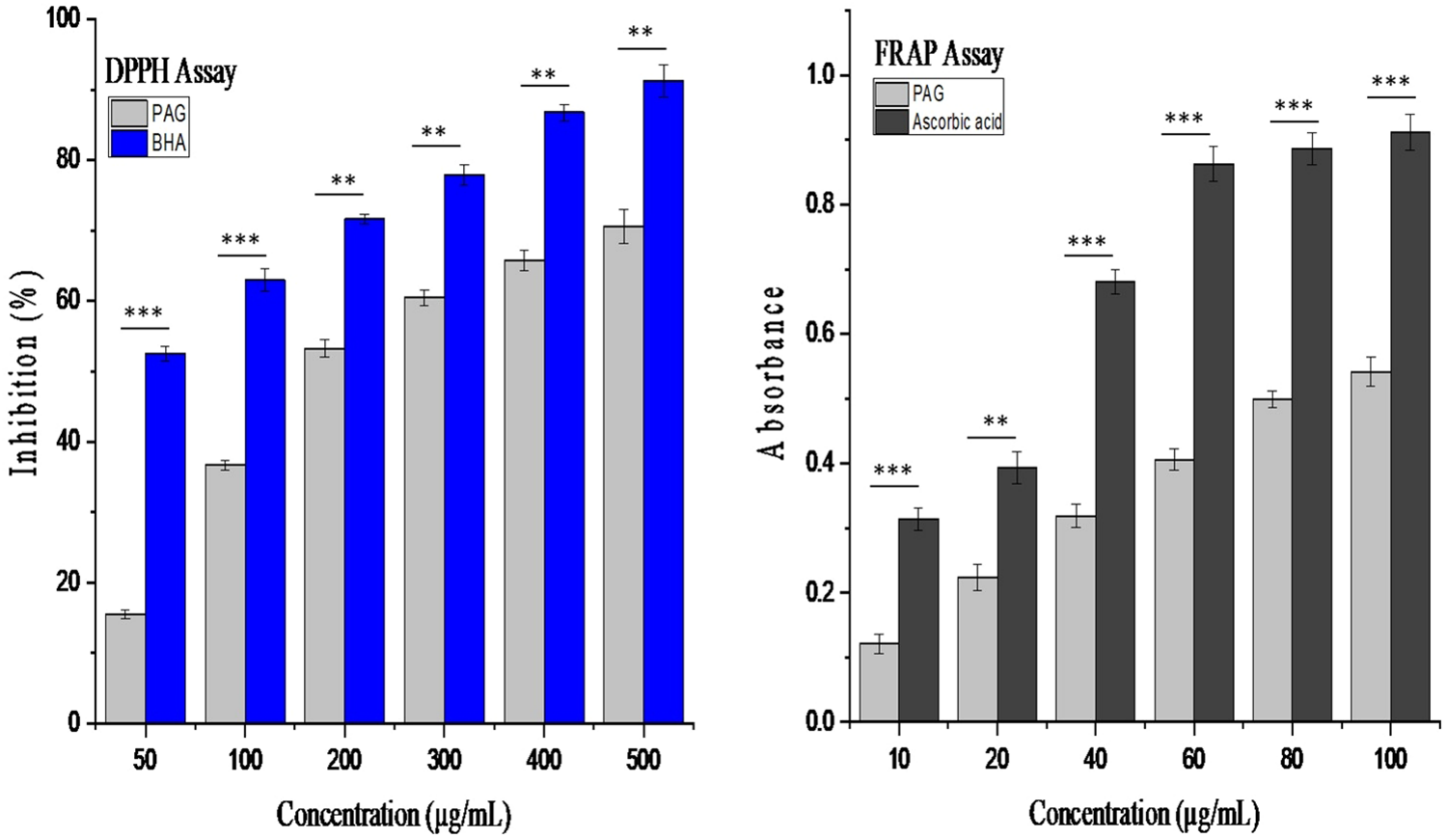
DDPH and FRAP assays of various concentrations of PAG and reference standard. All data are expressed as mean ± SD (n = 3), p < 0.001 (***) and p < 0.01 (**) on comparison with control group.

Polysaccharides are reported as an effective type of antioxidants^40^. So evaluation of a gum-based polymer for antioxidant potential is a worth doing activity. DPPH assay is one of the most adopted methods to test the antioxidant capacity of plant extracts^41^. DPPH is a free radical which shows a distinctive absorption peak at 517 nm. Decrease in absorbance value pinpoint towards radical scavenging potential of the sample^42^. It can be inferred from results shown in Fig. 6 that all concentrations of PAG exhibited DPPH radical scavenging activity. There was a consistent increase in inhibition percent with increasing concentration of gum polymer^27^. IC_50_ is the concentration of the sample where half of the maximum inhibition percent is obtained. It is the indicator of antioxidant activity of a substance. The IC_50_ of BHA and PAG was calculated from the graph between % Inhibition and concentration. The IC_50_ of the BHA and PAG solution was 48.35 μg/mL and 190.08 μg/mL respectively. In the FRAP assay, antioxidants of a sample reduce ferric (Fe^3+^) of ferric cyanide into ferrous ion (Fe^2+^). Higher values of absorbance of a sample solution indicate more reducing power^27,43^. Results shown in Fig. 6 in terms of absorbance illustrate that PAG have significant ability to terminate the chain reactions of radicals. Reducing power was increased as concentration was increased.

#### Antibacterial activity

**Figure 7:**
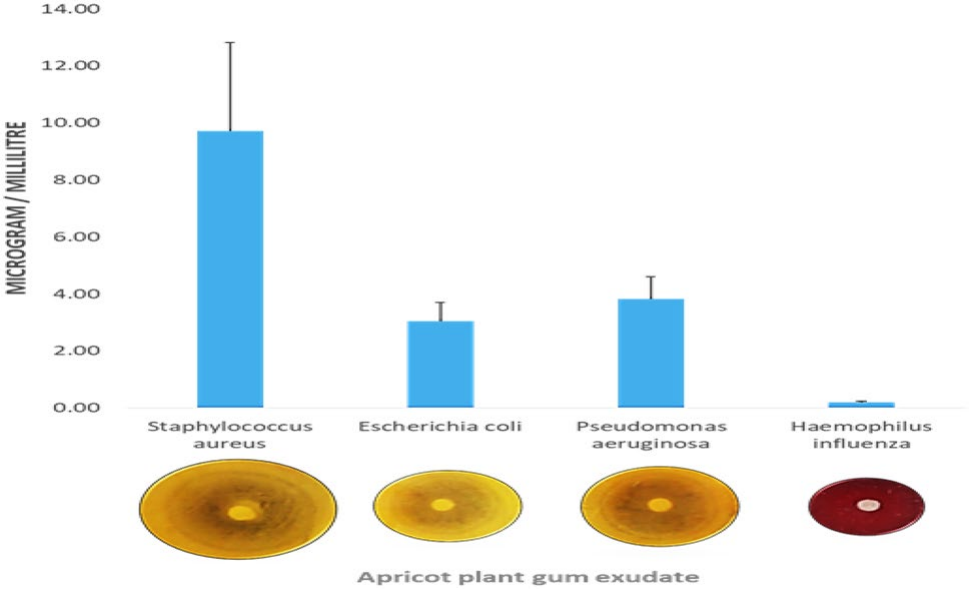
Antibacterial activity of PAG aqueous solution of PAG.

Uncontrolled and excessive use of marketed antibiotic has led towards a multiple drug resistance in microbes. Synthetic drugs have high manufacturing cost and may have undesirable side effects. So exploration for new odds with diverse origin and mechanisms is a topic of growing interest for investigators^44^. PAG showed antibacterial activity against all four selected strains. Results in terms of zones of inhibition (mm) are shown in Table 6. Highest value of inhibition zone was in case of *Staphylococcus aureus* and lowest for *Hemophilus influenza* as evident from the Table 6 and Fig. 7.

**Table 6:** Analysis with respect to test organism (Mean ± S.E of PAG in MIC).

Test organism	Mean ± S.E
*Staphylococcus aureus*	9.7222 ± 0.29361
*Escherichia coli*	5.0386 ± 0.48592
*Pseudomonas aeruginosa*	6.8194 ± 0.38666
*Hemophilus influenzae*	0.9953 ± 0.18064

#### Anti-nociceptive activity

##### Hot plate analysis

**Figure 8:**
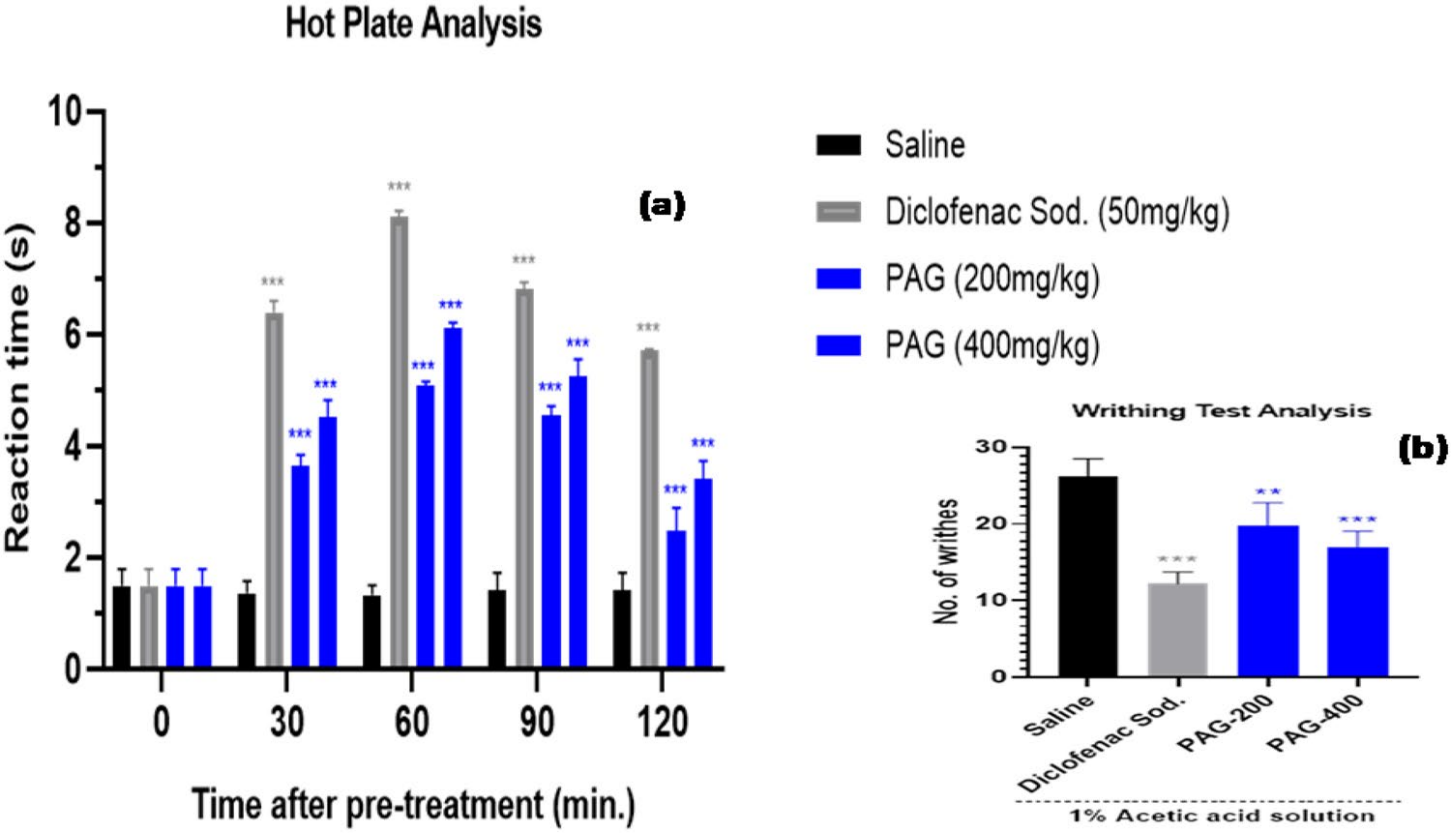
(**a**) Anti-nociceptive activity of PAG (200 mg/kg and 400 mg/kg) and Diclofenac sodium (50 mg/kg) evaluated using hot plate method. All data are expressed as mean ± SD (n = 5), p < 0.001 (***) on comparison with control (only saline treated) group. (**b**) Anti-nociceptive activity of PAG (200 mg/kg and 400 mg/kg) and Diclofenac sodium (50 mg/kg) evaluated using acetic acid induced writhing test. All data are expressed as mean ± SD (n = 5), p < 0.001 (***) and p < 0.01 (**) on comparison with control (only saline treated) group.

Anti-nociceptive (analgesic) activity of PAG was checked by using the hot plate analysis method. This method is mainly linked to evaluation of thermal stimulation effect or response to skin tissues and by measuring the licking time or jumping of animal used. According to Fig. 8a pre-treatment with PAG 200 mg/kg and 400 mg/kg) exposed significant anti-nociceptive effects by latency time prolongation. After 60 min of PAG treatment, maximum increase in the latency against thermal stimulation effect was 4.5 s (200 mg/kg) and 5.99 s (400 mg/kg) as compared to the control (1.58 s). Similar, but high effect around 8.2 s was noted with standard drug using as positive control i.e. diclofenac sodium (50 mg/kg) at 60 min. These findings were provided a hint that the anti-analgesic effect of PAG is linked to the central nervous system response of animals.

##### Writhing test analysis

Secondly, for evaluation of possible peripheral anti-nociceptive (analgesic) effect of PAG, a chemical stimulus based writhing test was applied. According to literature, it has been already reported that administration of the acetic acid solution decreased the writhing response. According to Fig. 8b, the pre-treatment with PAG (200 mg/kg and 400 mg/kg), significantly decreased the number of writhes (abdominal constrictions) with inhibition values of 25.9% at 200 mg/kg and 37.03% at 400 mg/kg of PAG pre-treatment. Similar effect was noted with standard drug using as positive control i.e. diclofenac sodium (50 mg/kg) with an inhibition rate of 54.9%, as compared to the control (only saline treated) group.

#### Anti-inflammatory activity

**Figure 9:**
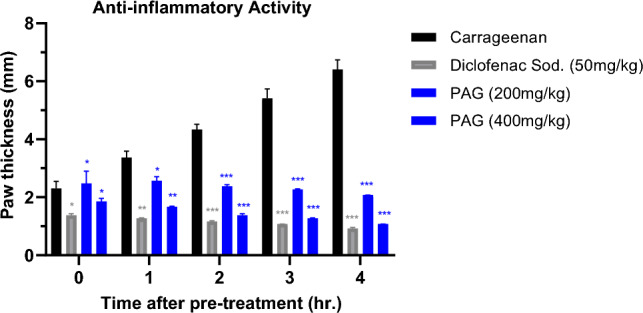
Anti-inflammatory effect of PAG (200 mg/kg and 400 mg/kg) and Diclofenac sodium (50 mg/kg) on carrageenan-induced mice paw edema. Each column represents mean ± SD (n = 5) per group. p < 0.001 (***), p < 0.01 (**) and p < 0.05 (*) on comparison with carrageenan treated control group.

PAG ameliorate inflammation as shown in the Fig. 9. Normal paw volume was prominently raised in all the groups after carrageenan solution injection. After 1 h of all treatments, significant reduction was observed with PAG doses of 200 mg/kg and 400 mg/kg in the carrageenan-induced paw edema during the subsequent 2–4 h of experiment. A similar anti-inflammatory profile was seen by the Diclofenac sodium (50 mg/kg) as a positive control during the 1–4 h of the study period.

#### Thrombolytic activity

Extensive studies are following the way to discover and modify natural components with antithrombotic activity to avoid drawbacks associated with synthetic drugs^33^. Plant polysaccharides shows diverse types of biological activities including antithrombotic activity^45^. Thrombolytic ability of PAG aqueous solution was check and results are depicted in Table 7. It can be inferred from the results that PAG showed a significant extent of thrombolytic activity as compared to standard Streptokinase. Extent of clot lysis was concentration dependent and increased with increasing concentration.

**Table 7:** Thrombolytic activity (%) of PAG aqueous solution.

Material	Thrombolytic activity (%)
PAG (%)	
0.2	2.15 ± 0.14
0.4	4.64 ± 0.47
0.6	8.39 ± 0.54
0.8	13.52 ± 1.22
1%	21.15 ± 1.58
Streptokinase	76.07 ± 1.55

#### Cytotoxicity analysis

**Figure 10:**
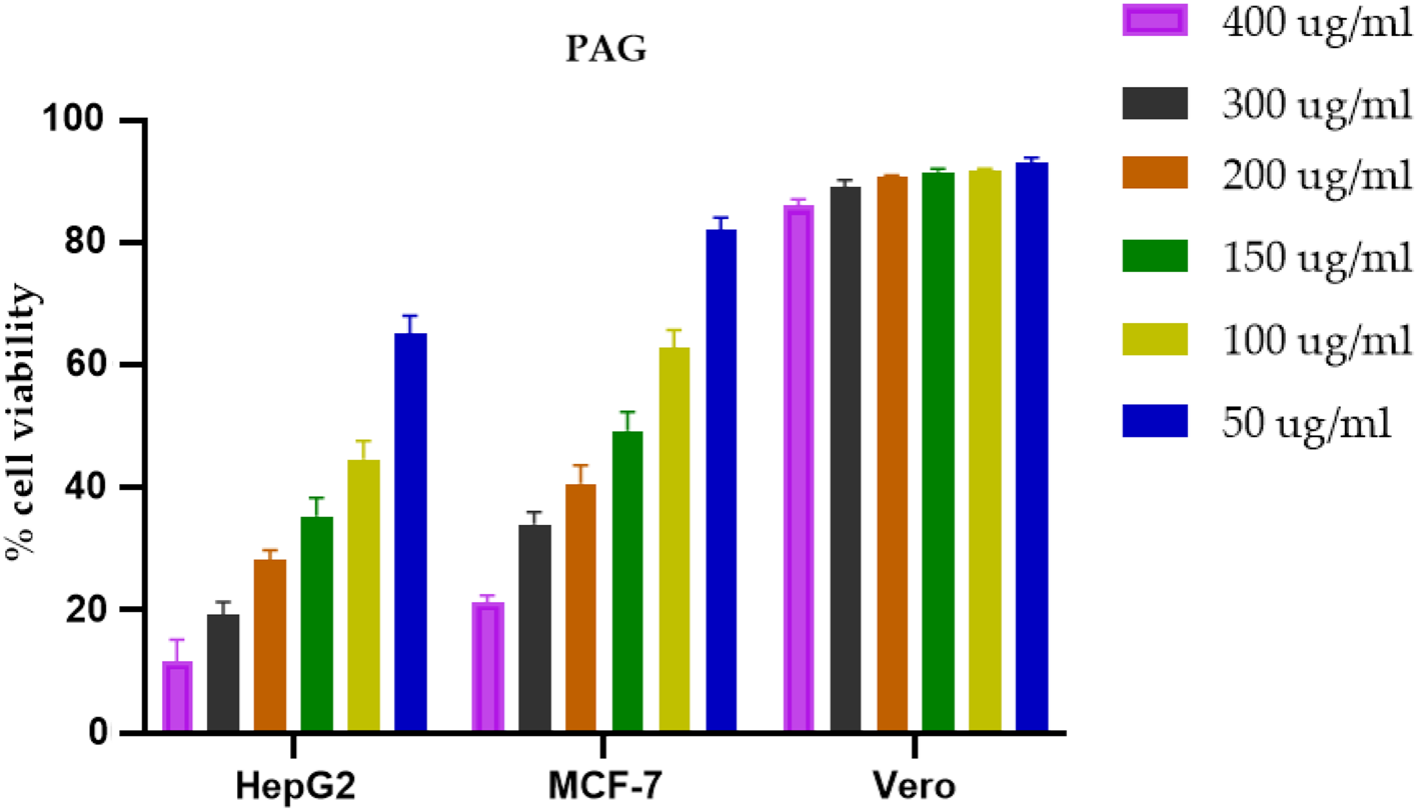
Quantitative cytotoxicity analysis of different concentrations of PAG on HepG2, MCF-7 cancer cells and Vero (normal cells) after 48 h of treatment.

The in vitro cytotoxic activity of PAG was investigated by MTT assay and carried out on normal cells (Vero cell lines) as well as cancer cell lines (HepG2 and MCF-7). The cell viability was high for Vero cells on treatment with all concentration of PAG, showing their non-toxic behavior and good biocompatibility for the normal cells. It indicates, normal cells will be less sensitive towards the anti-proliferating activity of PAG polymer. The purified PAG (Fig. 10) significantly inhibited cell proliferation in both HepG2 and MCF-7 cancer cell lines. Cytotoxic effect of PAG confirmed concentration dependent activity of PAG polymer (25–400 µg/mL). Results show that the anticancer efficacy of PAG was significant when compared with several literature reported activity of PAG^46^. However, further in vivo studies can be carried out for the complete characterization of anticancer efficacy of PAG.

## Conclusion

The RSM-CCD and ANN were used to get optimized conditions for extraction of PAG. A second-order quadratic model was achieved for the prediction of extraction yield (%) of PAG. The maximum yield of extraction predicted by both empirical models is at 25 °C, 6.4 pH, 6 h, and 1:20 PAG/water ratio which were close to experimental yield. LIBS spectra confirmed the presence calcium, potassium, magnesium, sodium, lithium, carbon, hydrogen, nitrogen and oxygen in extracted gum. Acute oral toxicity test confirmed that extracted PAG was non-toxic up to 2000 mg/kg in rabbits accompanied by high cytotoxic effect against HepG2 and MCF-7cells evaluated by MTT assay. PAG exhibited various pharmacological activities such as antioxidant, antibacterial, anti-nociceptive, anti-inflammatory, thrombolytic activities and anticancer activity. All these properties suggest that PAG is a promising natural candidate for various applications as described above for both food and pharmaceutical industry.

## Data Availability

All data obtained or analyzed during study is reported in the article.
